# Artery of Adamkiewicz: a meta-analysis of anatomical characteristics

**DOI:** 10.1007/s00234-019-02207-y

**Published:** 2019-04-27

**Authors:** Dominik Taterra, Bendik Skinningsrud, Przemysław A. Pękala, Wan Chin Hsieh, Roberto Cirocchi, Jerzy A. Walocha, R. Shane Tubbs, Krzysztof A. Tomaszewski, Brandon Michael Henry

**Affiliations:** 1 International Evidence-Based Anatomy Working Group, Kraków, Poland; 2 Department of Anatomy, Jagiellonian University Medical College, 12 Kopernika Street, 31-034 Kraków, Poland; 3 First Faculty of Medicine, Charles University, Prague, Czech Republic; 4 Department of Surgical Sciences, Radiology and Dentistry, University of Perugia, Perugia, Italy; 5 Seattle Science Foundation, Seattle, WA USA; 6 Faculty of Medicine and Health Sciences, Andrzej Frycz Modrzewski KrakowUniversity, Kraków, Poland

**Keywords:** Adamkiewicz artery, Anatomy, Great anterior radiculomedullary artery, Thoracoabdominal aneurysm, Aortic aneurysm

## Abstract

**Purpose:**

The artery of Adamkiewicz (AKA) provides the major blood supply to the anterior thoracolumbar spinal cord and iatrogenic injury or inadequate reconstruction of this vessel during vascular and endovascular surgery can result in postoperative neurological deficit due to spinal cord ischemia. The aim of this study was to provide comprehensive data on the prevalence and anatomical characteristics of the AKA.

**Methods:**

An extensive search was conducted through the major electronic databases to identify eligible articles. Data extracted included study type, prevalence of the AKA, gender, number of AKA per patient, laterality, origin based on vertebral level, side of origin, morphometric data, and ethnicity subgroups.

**Results:**

A total of 60 studies (*n* = 5437 subjects) were included in the meta-analysis. Our main findings revealed that the AKA was present in 84.6% of the population, and patients most frequently had a single AKA (87.4%) on the left side (76.6%) originating between T8 and L1 (89%).

**Conclusion:**

As an AKA is present in the majority of the population, caution should be taken during vascular and endovascular surgical procedures to avoid injury or ensure proper reconstruction. All surgeons operating in the thoracolumbar spinal cord should have a thorough understanding of the anatomical characteristics and surgical implications of an AKA.

**Electronic supplementary material:**

The online version of this article (10.1007/s00234-019-02207-y) contains supplementary material, which is available to authorized users.

## Introduction

**Fig. 1:**
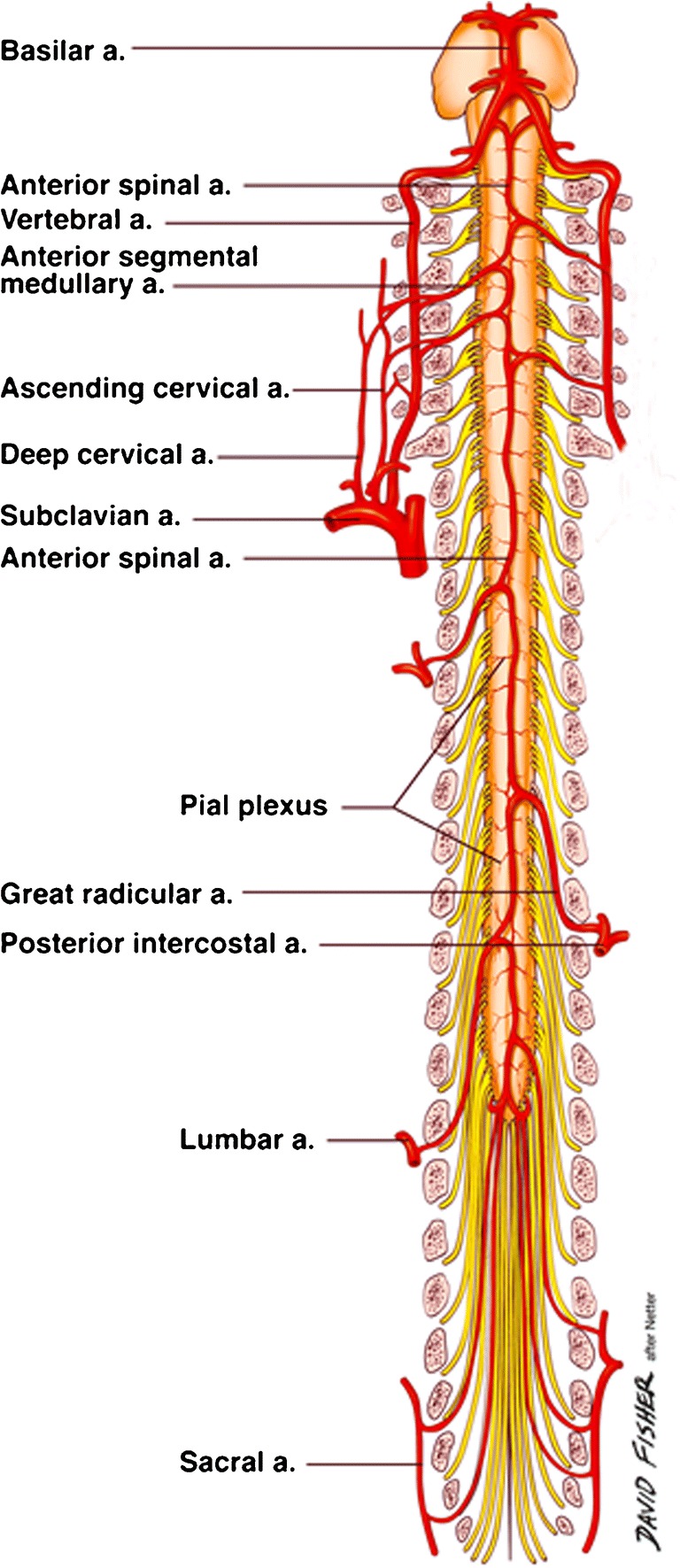
Vasculature of the spinal cord—the artery of Adamkiewicz (great radicular a.)

**Fig. 2:**
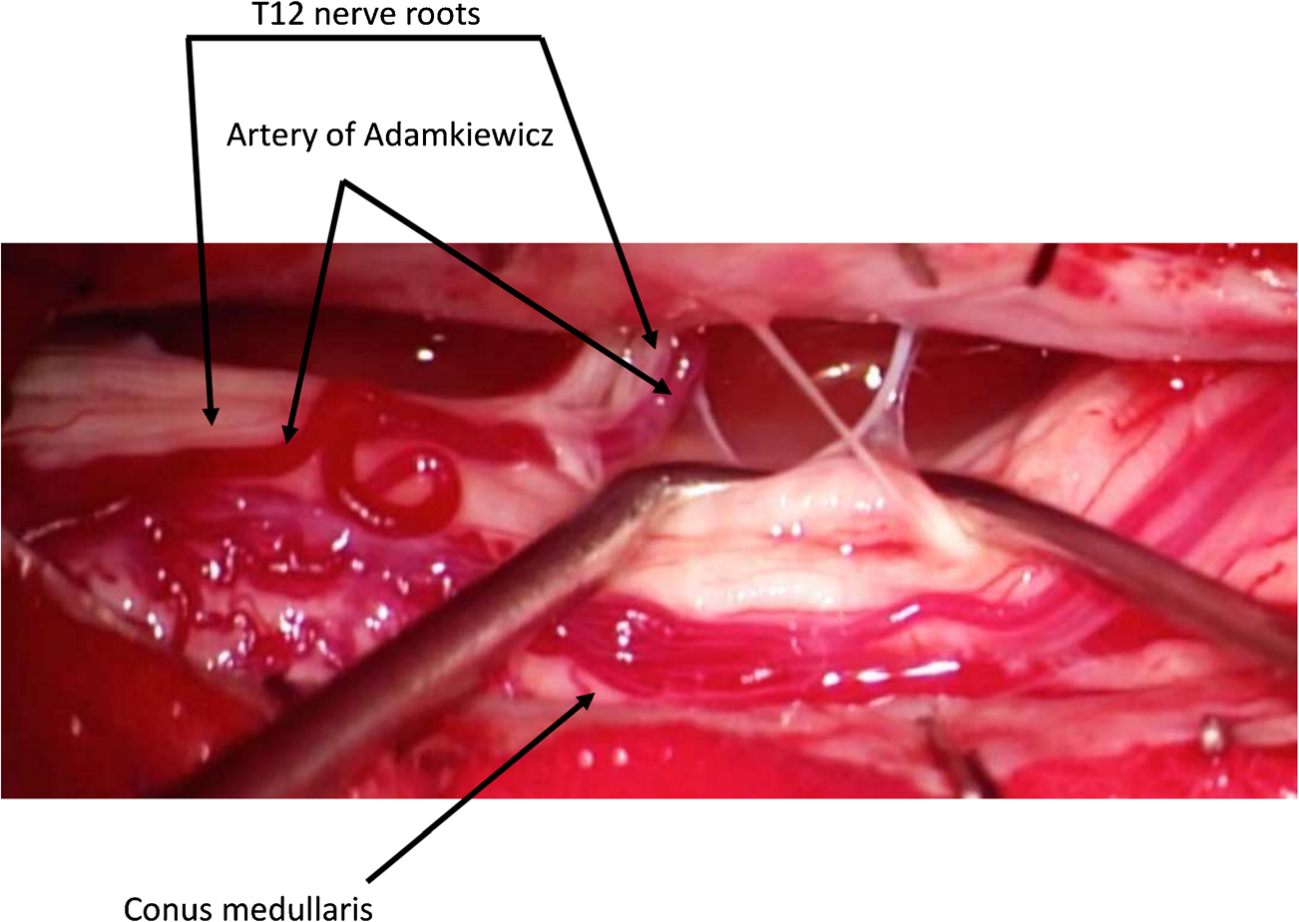
Intraoperative image of the artery of Adamkiewicz

The artery of Adamkiewicz (AKA), also known as the great anterior radiculomedullary artery, is a major artery that joins the anterior spinal artery in the lower one-third of the spinal cord (Fig. 1) [1]. Because of its large role in feeding the spinal cord, many reports have stressed the importance of reattaching the intercostal or lumbar arteries to the AKA in the event of spinal cord ischemia following vascular and endovascular surgery (Fig. 2). Identification of the AKA preoperatively helps surgeons to determine the appropriate range of aortic lesions that require graft replacement [2]. Therefore, accurate localization and detailed anatomical knowledge of the AKA are important when planning surgical and interventional radiological treatments of thoracoabdominal diseases and spinal lesions in order to help reduce the risk of postoperative ischemic spinal complications and paraplegia.

**Fig. 3:**
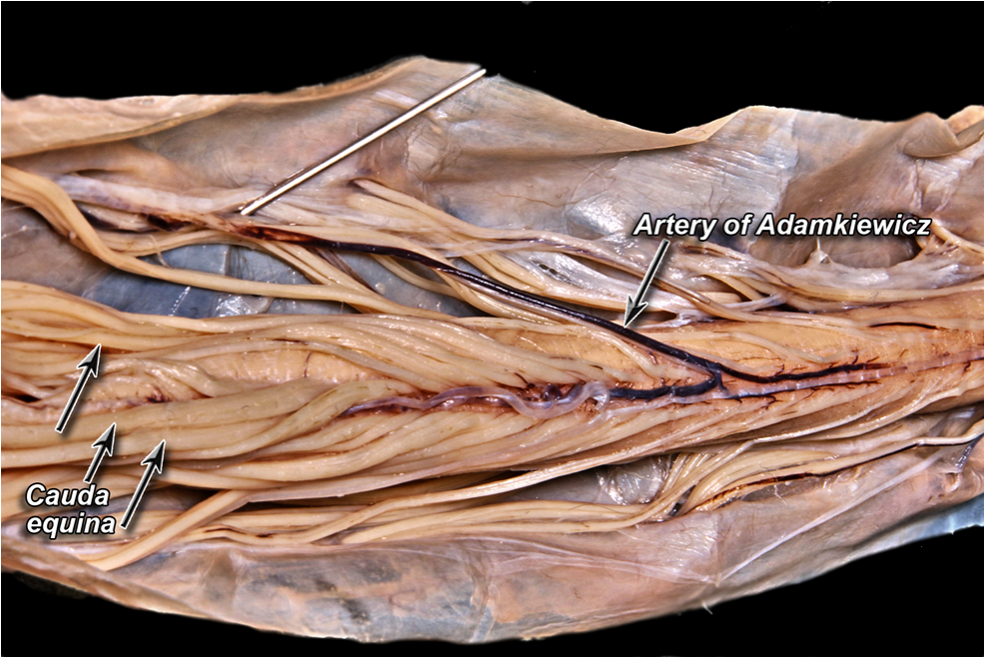
Cadaveric dissection of the artery of Adamkiewicz

The AKA is the most dominant anterior radiculomedullary artery and is responsible for the arterial blood supply to the spinal cord from T8 to the conus medullaris [3]. Its origin is highly variable and extends from the mid-thoracic level to the lumbar levels, including the bilateral T3-T12 intercostal arteries [4] and L1-L4 lumbar arteries [5]. It typically arises from the T8–L1 neural foramina [6] from the left intercostal or lumbar arteries [7]. The AKA has a diameter of 0.8–1.3 mm, and the distal portion of this artery, together with the anterior spinal artery, forms a characteristic “hairpin” turn [8] (Fig. 3). Various techniques have been devised to preoperatively identify the location and anatomy of this artery. Such techniques include computed tomography angiography (CTA), magnetic resonance angiography (MRA), and digital subtraction angiography (DSA), with the latter considered the gold standard [9].

The most important cause of injury to the AKA is iatrogenic, and in part, this is a factor of the high degree of variability in the anatomical location of this artery [10]. Preoperative AKA identification and its subsequent reconstruction or preservation may aid in reducing the incidence of postoperative neurological deficits and improving the outcomes of thoracolumbar surgical procedures. To this end, the aim of this study was to provide comprehensive data on the prevalence and anatomical characteristics of the AKA.

## Materials and methods

### Search strategy

A search of all major electronic databases (PubMed, EMBASE, ScienceDirect, China National Knowledge Infrastructure (CNKI), SciELO, BIOSIS, and Web of Science) was performed in order to identify potential articles. The following search terms were employed: artery of Adamkiewicz, arteria radicularis magna (ARM), radicularis magna, great radicular artery of Adamkiewicz, major anterior segmental medullary artery, great anterior segmental medullary artery, artery of the lumbar enlargement, arteria radicularis anterior magna, and great anterior radiculomedullary artery. A search through the references of the initially selected articles was conducted to identify any potential studies that were omitted. The authors adhered to the Preferred Reporting Items for Systematic Reviews and Meta-Analyses (PRISMA) guidelines throughout this meta-analysis (Supplement 1).

### Eligibility assessment

An eligibility assessment was conducted by two independent reviewers. Studies were included in this meta-analysis if they (1) provided complete data on the prevalence of the AKA or (2) provided data on the anatomy of AKA. The following exclusion criteria were employed: case, case-series, conference abstracts, letters to editors, and studies not published in peer-reviewed journals. Studies that were originally published in languages other than English were translated by medical professionals who are fluent both in English and the original language of the manuscript. All differences of opinion among the reviewers concerning the eligibility of the studies were resolved by consensus through consultation with the author of the respective study.

### Data extraction

Two reviewers carried out data extraction independently. The following data was extracted: publication year, country of origin, study type (cadaveric, CTA, MRA, DSA), prevalence data of AKA, number of AKAs per patient, laterality of the AKA, origin of the AKA based on the vertebral level, side of origin, and morphometric data. In cases of incomplete data, the authors of the original articles were contacted for clarification.

### Quality assessment

The AQUA tool [11] was used by the reviewers to evaluate quality and reliability of the included studies. In brief, the tool was devised to probe for potential risk of bias. Five domains were evaluated in the analysis: (1) objective(s) and subject characteristics, (2) study design, (3) methodology characterization, (4) descriptive anatomy, and (5) reporting of results; and each domain was categorized as either of “Low,” “High,” or “Unclear” risk of bias. Decision was made that a “No” answer in whichever signaling question within each of the categories arbitrated the domain to be of “High” risk of bias, whereas all answers “Yes” suggested that it presented a “Low” risk of bias. “Unclear” option was chosen when the study with incoherent data did not permit for a clear scrutiny.

### Statistical analysis

The prevalence analysis was conducted using MetaXL version 5.8 by EpiGear Pty Ltd. (Wilston, Queensland, Australia). Morphometric analysis using Comprehensive Meta-Analysis version 3.3 yielded the pooled mean diameter of the AKA. Single and multi-categorical pooled prevalence rates were calculated using a random effects model. Heterogeneity was assessed using a chi-squared test and the *I*^2^ statistic. For the *I*^2^ statistic, the values of 0–40% indicated that heterogeneity might not be important; values of 30–60% could indicate moderate heterogeneity; values of 50–90% could indicate substantial heterogeneity; and values of 75–100% indicated considerable heterogeneity. A *p* value below 0.10 for Cochran’s Q suggested significant heterogeneity [12].

An analysis of the subgroups was conducted to determine the source of heterogeneity. The difference between the groups was considered to be insignificant if the confidence intervals (CIs) of specific rates overlapped [13]. Subgroups according to study type, gender, and geographical location were analyzed.

## Results

### Study identification and characteristics of included studies

**Fig. 4:**
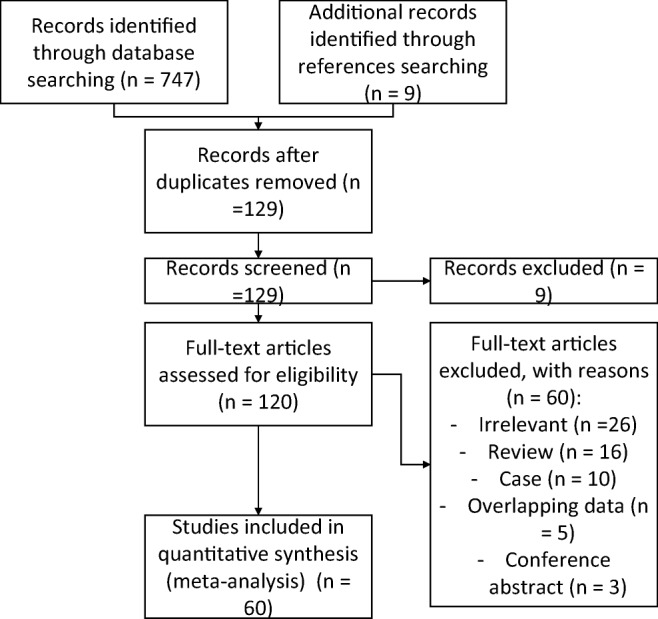
Flow diagram of included studies

The study identification process is presented in Fig. 4. An initial search yielded 747 entries. After thorough analysis, 627 entries were excluded. In total, 120 articles were analyzed, and 60 studies were included in this meta-analysis.

The characteristics of the included studies are presented in Table 1. A total of 60 studies (4317 subjects with AKA) published between 1989 [14] and 2017 [15] were included [1–10, 14–62]. The studies originated from North America, Asia and Europe, and from ten different countries.

**Table 1:** Characteristics of included studies

Study	Country	Type of study	Number of subjects	% prevalence of AKA (no. of subjects with AKA)
Alleyne 1998	USA	Cadaveric	10	90.0 (9)
Amako 2011	Japan	CTA	110	100.0 (110)
Bachet 1996	France	CTA	36	77.8 (28)
Backes 2008	Netherlands	MRA	85	100.0 (85)
Biglioli 2004	Italy	Cadaveric	51	100.0 (51)
Bley 2010	Germany	MRA	68	88.2 (60)
Boll 2006	USA	MDCT angiography	100	100.0 (100)
Bowen 1996	USA	MRA	6	100.0 (6)
Champlin 1994	USA	DSA	61	32.8 (20)
Charles 2011	France	DSA	100	96.0 (96)
Fanous 2015	USA	DSA	34	70.6 (24)
Fereshetian 1989	USA	DSA	12	75.0 (9)
Furukawa 2010	Japan	CTA	37	100.0 (37)
Gailloud 2013	USA	DSA	50	92.0 (46)
Guzinski 2017	Poland	MSCT	200	21.5 (43)
Heinemann 1998	Germany	DSA	46	65.2 (30)
Hyodoh 2005	Japan	MRA	50	84.0 (42)
Hyodoh 2007	Japan	MRA (double subtraction maximum intensity projection)	170	82.4 (140)
Hyodoh 2009	Japan	MRA	82	81.7 (67)
Jaspers 2007	Netherlands	MRA	20	100.0 (20)
Kawaharada 2002	Japan	MRA	40	72.5 (29)
Kawaharada 2004	Japan	MRA	120	82.5 (99)
Kawaharada 2007	Japan	MRA	83	85.5 (71)
Kieffer 1989	France	Arteriography	45	88.9 (40)
Kieffer 2002	France	Arteriography	480	87.3 (419)
Koshino 1999	Japan	Cadaveric	102	88.2 (90)
Kovacs 2009	Germany	CT	51	70.6 (36)
Kroszczynski 2013	USA	Cadaveric	24	95.8 (23)
Kudo 2003	Japan	MDCT	19	68.4 (13)
Matsuda 2010	Japan	MRA and CTA	50	94.0 (47)
Matsuda 2010a	Japan	MRA and CTA	60	80.0 (48)
Melissano 2009	Italy	MDCT	67	67.2 (45)
Mordasini 2012	Switzerland	MRA	24	83.3 (20)
Morishita 2003	Japan	Cadaveric	55	100.0 (55)
Murthy 2010	USA	Spinal angiography	248	46.4 (115)
Nakayama 2008	Japan	CTA	80	56.3 (45)
Nijenhuis 2004	Netherlands	MRA	8	100.0 (8)
Nijenhuis 2007	Netherlands	MRA and CTA	39	100.0 (39)
Nijenhuis 2007a	Netherlands	MRA	60	100.0 (60)
Nishida 2014	Japan	CT	33	75.8 (25)
Nishii 2013	Japan	CTA	160	81.9 (131)
Nojiri 2007	Japan	CTA	27	100.0 (27)
Ogino 2006	Japan	MRA	92	70.7 (65)
Ou 2007	France	CTA	40	95.0 (38)
Polaczek 2014	Poland	Cadaveric	28	100.0 (28)
Rodriguez-Baeza 1991	Spain	Cadaveric	30	100.0 (30)
Schurink 2007	Netherlands	MRA	9	100.0 (9)
Sukeeyamonon 2010	Thailand	MDCT angiography	73	71.2 (52)
Takagi 2015	Japan	MRA and MDCTA	117	89.7 (105)
Takase 2002	Japan	MDCT	70	90.0 (63)
Takase 2007	Japan	MDCT	10	90.0 (9)
Tanaka 2016	Japan	MRA and CTA	1252	87.5 (1096)
Uotani 2008	Japan	CTA	32	78.1 (25)
Utsunomiya 2008	Japan	CTA	80	62.5 (50)
Williams 1991	USA	Retrograde femoral artery catherization	47	55.3 (26)
Yamada 2000	Japan	MRA	26	69.2 (18)
Yingbin 2013	China	MDCT	217	55.8 (121)
Yoshioka 2003	Japan	MRA and CTA	30	90.0 (27)
Yoshioka 2006	Japan	MRA and CTA	30	96.7 (29)
Zhao 2009	China	MDCTA	51	35.3 (18)

### Quality assessment

The majority of studies included in this meta-analysis, evaluated by the AQUA tool, revealed domain one (objective(s) and subject characteristics) and domain three (methodology characterization) to be at “High” risk of bias, owing to missing demographic data of the research group and no information regarding experience of the researchers. All studies had a “Low” risk of bias found in domain two (study design) and domain five (reporting of results), and almost all studies had a “Low” risk of bias found in domain four (descriptive anatomy). The AQUA tool evaluation can be found in Table 2.

**Table 2:** The AQUA tool—tabular display

Study	Risk of bias				
	Objective(s) and study characteristics	Study design	Methodology characterization	Descriptive anatomy	Reporting of results
Alleyne 1998	High	Low	High	Low	Low
Amako 2011	Low	Low	High	Low	Low
Bachet 1996	High	Low	High	High	Low
Backes 2008	High	Low	High	Low	Low
Biglioli 2004	Low	Low	High	High	Low
Bley 2010	Low	Low	High	Low	Low
Boll 2006	High	Low	High	High	Low
Bowen 1996	High	Low	High	Low	Low
Champlin 1994	High	Low	High	Low	Low
Charles 2011	High	Low	High	Low	Low
Fanous 2015	High	Low	High	Low	Low
Fereshetian 1989	High	Low	High	Low	Low
Furukawa 2010	High	Low	High	Low	Low
Gailloud 2013	High	Low	High	Low	Low
Guzinski 2017	High	Low	High	Low	Low
Heinemann 1998	High	Low	High	Low	Low
Hyodoh 2005	High	Low	High	High	Low
Hyodoh 2007	High	Low	High	Low	Low
Hyodoh 2009	High	Low	High	High	Low
Jaspers 2007	High	Low	High	Low	Low
Kawaharada 2002	High	Low	High	Low	Low
Kawaharada 2004	High	Low	High	Low	Low
Kawaharada 2007	High	Low	High	Low	Low
Kieffer 1989	High	Low	High	High	Low
Kieffer 2002	High	Low	High	Low	Low
Koshino 1999	High	Low	High	Low	Low
Kovacs 2009	High	Low	High	Low	Low
Kroszczynski 2013	High	Low	High	Low	Low
Kudo 2003	High	Low	High	Low	Low
Matsuda 2010	High	Low	High	High	Low
Matsuda 2010a	High	Low	High	High	Low
Melissano 2009	High	Low	Low	High	Low
Mordasini 2012	High	Low	Low	High	Low
Morishita 2003	High	Low	High	Low	Low
Murthy 2010	Unclear	Low	High	Low	Low
Nakayama 2008	High	Low	Low	Low	Low
Nijenhuis 2004	High	Low	Unclear	Low	Low
Nijenhuis 2007	High	Low	High	Low	Low
Nijenhuis 2007a	High	Low	High	Low	Low
Nishida 2014	High	Low	Low	High	Low
Nishii 2013	High	Low	Low	High	Low
Nojiri 2007	High	Low	High	Low	Low
Ogino 2006	High	Low	High	High	Low
Ou 2007	High	Low	Unclear	High	Low
Polaczek 2014	High	Low	High	Low	Low
Rodriguez-Baeza 1991	High	Low	High	Low	Low
Schurink 2007	High	Low	High	Low	Low
Sukeeyamonon 2010	High	Low	Low	High	Low
Takagi 2015	High	Low	Low	Low	Low
Takase 2002	High	Low	High	Low	Low
Takase 2007	High	Low	High	High	Low
Tanaka 2016	High	Low	High	Low	Low
Uotani 2008	High	Low	High	Low	Low
Utsunomiya 2008	High	Low	High	Low	Low
Williams 1991	High	Low	High	Low	Low
Yamada 2000	High	Low	High	Low	Low
Yingbin 2013	High	Low	High	Low	Low
Yoshioka 2003	High	Low	High	Low	Low
Yoshioka 2006	High	Low	High	Low	Low
Zhao 2009	High	Low	High	Low	Low

### Prevalence of the artery of Adamkiewicz

A total of 60 studies (*n* = 5437 subjects) reported data on the prevalence of the AKA. The pooled prevalence estimate (PPE) of the AKA was 84.6% (95% CI 79.7–89.0) (Table 3).

**Table 3:** Overall prevalence of AKA

Subgroup		Number of studies (number of subjects)	Pooled prevalence of AKA: % (95% CI)	*I*^2^ % (95% CI)	Cochran’s *Q*, *p* value
Overall		60 (5437)	84.6 (79.7–89.0)	95.3 (94.5–95.9)	< 0.001
Gender	Males	15 (515)	93.7 (83.3–100.0)	94.0 (91.6–95.7)	< 0.001
	Females	14 (345)	90.4 (68.9–100.0)	96.4 (95.2–97.4)	< 0.001
Type of study	Cadaveric	7 (300)	97.5 (92.4–100.0)	72.2 (38.8–87.1)	0.001
	CTA	9 (602)	88.1 (74.0–97.6)	94.4 (91.4–96.4)	< 0.001
	MRA	16 (943)	88.3 (81.9–93.4)	85.1 (77.3–90.3)	< 0.001
	DSA	6 (303)	75.4 (49.1–94.9)	94.9 (91.2–97.0)	< 0.001
Country of origin	Japan	27 (3017)	85.3 (81.0–89.2)	87.5 (83.0–90.8)	< 0.001
	USA	10 (592)	79.5 (57.0–95.7)	96.3 (94.7–97.4)	< 0.001
	France	5 (701)	89.8 (83.8–94.6)	69.0 (20.4–87.9)	0.012
	Netherlands	6 (221)	99.4 (98.2–100.0)	0.0 (0.0–0.0)	0.972

The subgroup analysis of gender differences showed that the AKA was slightly more prevalent in males (93.7% [95% CI 83.3–100.0]) than females (90.4% [95% CI 68.9–100.0]), although not significantly.

Seven cadaveric studies (*n* = 300) yielded the highest PPE of the AKA (97.5% [95% CI 92.4–100.0]) among the different study types. This was followed by MRA, CTA, and DSA studies with PPEs of 88.3%, 88.1%, and 75.4%, respectively (Table 3).

The subgroup analysis of geographical origin showed that the AKA was most prevalent in the Netherlands, with a PPE of 99.4% (95% CI 98.2–100.0); France with a PPE of 89.8% (95% CI 83.8–94.6); and Japan, with a PPE of 85.3 (95% CI 81.0–89.2). It was least prevalent in the USA, with a PPE of 79.5% (95% CI 57.0–95.7).

### Number of arteries of Adamkiewicz per patient

An analysis of 20 studies (*n* = 1329 subjects with AKAs) showed that the majority of patients (87.4% [95% CI 83.4–91.9]) had one AKA. Patients presented with two AKAs in 11.3% (95% CI 7.5–15.8) of cases, three AKAs in 0.8% (95% CI 0.0–2.5) of cases, and four AKAs in 0.5% (95% CI 0.0–1.6) of cases.

In patients with two AKAs, the majority (73.3% [95% CI 47.3–93.4]) presented unilaterally as duplications. A total of 26.7% (95% CI 6.6–52.7; *I*^2^ 66.2%, 95% CI 12.0–87.0; *p* = 0.019) of patients with two AKAs had bilateral configuration.

### Origin of the artery of Adamkiewicz

A total of 56 studies (*n* = 3316 patients with AKA) analyzed the side of origin of AKA. The results showed that 76.6% (95% CI 73.2–79.9) of AKAs originated from the left side, while 23.4% (95% CI 20.1–26.8; *I*^2^ 78.5%, 95% CI 72.5–83.2; *p* < 0.001) from the right side. The analysis of 43 studies (*n* = 2834 patients with AKA) showed that 89% of arteries originated between T8 and L1 (Table 4). AKA most frequently originated at the level of T9 with PPE of 22.2% (95% CI 18.9–25.4), followed by T10 and T11 with PPE of 21.7% (95% CI 18.5–25.0) and 18.7% (95% CI 15.6–21.8), respectively.

**Table 4:** Origin of AKA (vertebral levels)

Number of studies (number of subjects with AKA)	T3: % (95% CI)	T4: % (95% CI)	T5: % (95% CI)	T6: % (95% CI)	T7: % (95% CI)	T8: % (95% CI)	T9: % (95% CI)	T10: % (95% CI)	T11: % (95% CI)	T12: % (95% CI)	L1: % (95% CI)	L2: % (95% CI)	L3: % (95% CI)	L4: % (95% CI)	L5: % (95% CI)	I^2^: % (95% CI)	Cochran’s Q, p value
43 (2834)	0.5 (0.1–1.3)	0.7 (0.2–1.6)	0.8 (0.2–1.7)	0.8 (0.2–1.8)	2.2 (1.2–3.5)	7.3 (5.3–9.4)	22.2 (18.9–25.4)	21.7 (18.5–25.0)	18.7 (15.6–21.8)	12.2 (9.7–14.8)	6.9 (5.0–9.0)	3.8 (2.4–5.5)	1.1 (0.4–2.1)	0.5 (0.1–1.3)	0.5 (0.1–1.2)	74.7 (66.0–81.2)	< 0.001

### Continuity of the artery of Adamkiewicz

A total of seven studies (*n* = 375 patients with AKAs) were included in an analysis of the continuity of the AKA from the aorta to the anterior spinal artery. The results showed that AKA continued from the aorta to the anterior spinal artery in 71.3% of patients (95% CI 45.8–91.6; *I*^2^ 95.6%, 95% CI 92.8–97.2; *p* < 0.001).

### Morphometric analysis of the artery of Adamkiewicz

Five studies (*n* = 324 patients with AKA) analyzed the morphometric data of the AKA. The analysis showed a pooled mean diameter of 1.09 mm (95% CI 0.69–1.50; *I*^2^ 36.2%; *p* < 0.001).

## Discussion

Because the AKA originates from the lumbar arteries, it may be prudent to preserve the blood flow from the lumbar arteries when a thoracoabdominal aortic repair is planned [5, 63]. Concomitant or previous abdominal aortic repair and extensive thoracic aorta exclusion by means of multiple stent grafts are associated with a significantly higher risk of paraplegia [64]. After the interruption of most of the intercostal and lumbar arteries, the residual collateral blood supply is marginal, and in some cases, the spinal cord may become extremely prone to injury due to arterial hypotension or low cardiac output from any cause [65]. During aortic repair, preservation, reattachment, or reconstruction of the intercostal or lumbar arteries can maintain the blood supply to the spinal cord [66, 67]. Depending on the number of intercostal or lumbar arteries that require reconstruction, the ischemic duration may be prolonged during reconstruction. In our study, in patients with an AKA present, 11.3% had two AKAs, with bilateral AKAs present in 26.7% of these patients. The preoperative identification of the AKA and its anatomical characteristics allows for superior surgical planning, such that the surgical time and postoperative spinal complication risk are decreased [31]. Therefore, AKA identification is of interest for surgeons aiming to reconstruct intercostal or lumbar arteries in order to prevent postoperative spinal ischemic complications [3].

With respect to the continuity between the radicular arteries (including the AKA) and the anterior spinal arteries, the AKA continued from the aorta to the anterior spinal artery in 71.3% of the patients in our study. When this continuity is present, blood may drain away from the spinal cord through the anterior spinal arteries and the radicular arteries, acting as stealing channels by rerouting the blood to be distal to an aortic obstruction [5]. During aortic cross-clamping, back-bleeding from the ostia of the posterior intercostal and lumbar arteries may be a clinical manifestation of such rerouting of blood when continuity between the AKA and the anterior spinal arteries is present. This steal phenomenon may further worsen spinal cord ischemia, causing irreversible neurological injuries if the ischemia time is longer than 20 to 30 min [68].

**Fig. 5:**
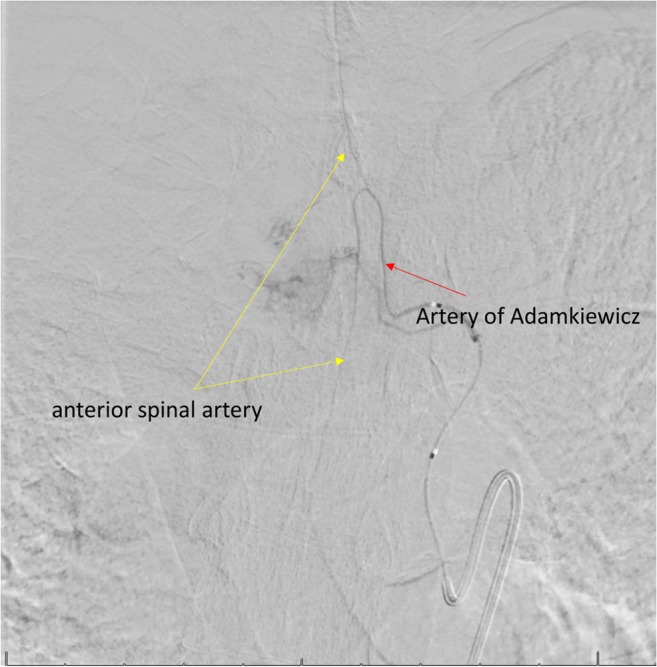
Digital subtraction angiography image of the artery of Adamkiewicz from left T4 radicular artery injection

**Fig. 6:**
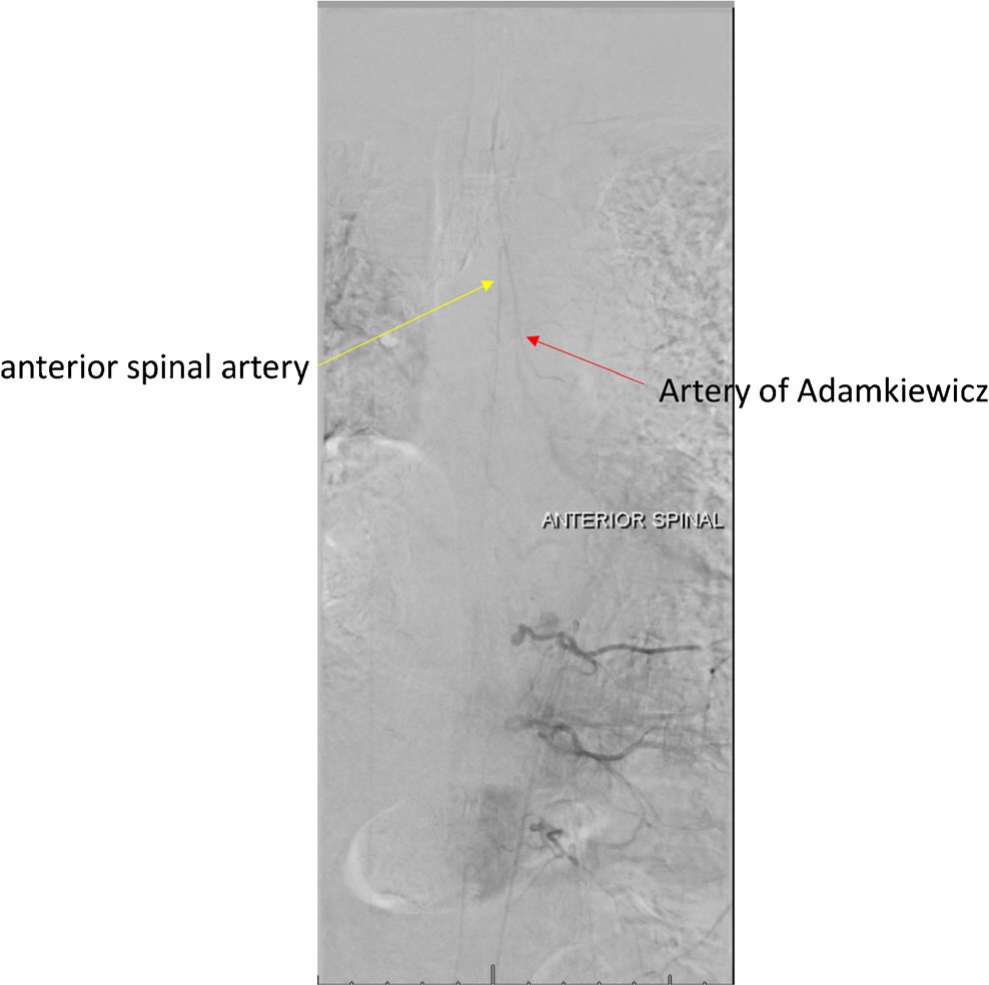
Digital subtraction angiography image of the artery of Adamkiewicz from left T8 radicular artery injection

**Fig. 7:**
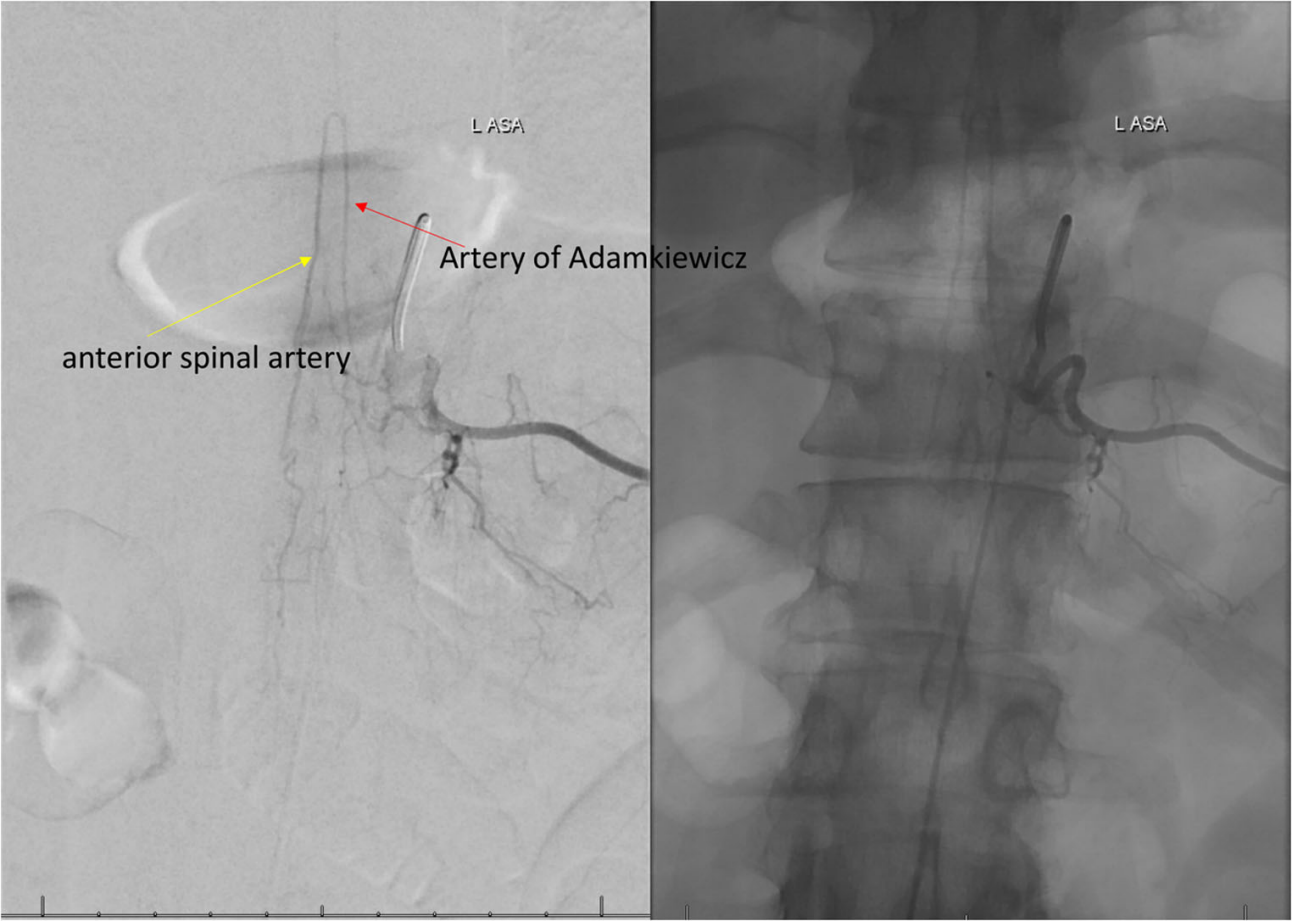
Digital subtraction angiography image of the artery of Adamkiewicz from left T11 radicular artery injection

The detection of the AKA can be difficult because of the various possible levels of origins of the artery, its small size, the amount of time needed to obtain the angiogram, and complications that can occur during surgical procedures [14, 26]. In our study, the pooled mean diameter of the AKA was 1.09 mm. Various techniques have been devised to preoperatively identify the location and anatomy of the AKA, such as CTA [55], MRA [7], and DSA [9]. These techniques can be used to identify both the level and the laterality of the artery, which can affect a surgeon’s approach to an aneurysm or spinal lesion. We have included three DSA images with injected contrast into left radicular artery at the level of T4 (Fig. 5), T8 (Fig. 6), and T11 (Fig. 7). In our meta-analysis, cadaveric studies had the highest prevalence of an AKA (97.5%), and among the different imaging modalities, MRA and CTA had the highest prevalence rates (88.3% and 88.1%, respectively), while DSA had the lowest prevalence rate (75.4%). In spite of its apparent success in detecting an AKA, MRA has been shown to be inferior to DSA in terms of evaluating vessel continuity, sharpness, and background homogeneity [7]. Furthermore, compared with CTA, a more limited field of view is a major disadvantage of MRA [61]. As a result, MRA may fail to depict the clinically important collateral vessels to the AKA in some patients, when a collateral source is the internal thoracic artery or the thoracodorsal artery [69]. Despite DSA studies reporting a lower prevalence rate of the AKA than MRA and CTA in our meta-analysis, DSA remains the “gold standard” for identifying spinal cord vasculature as it is both safe and efficient [9]. A possible reason for this discrepancy could be the small number of patients included in our DSA analysis as compared to the number of patients included in our MRA and CTA analyses.

Future studies should examine the blood supply and the collateral circulation of the spinal cord in the presence of degenerative atherosclerotic or dissecting aneurysm, or after a surgical or endovascular aortic procedure. In these patients, the disease and the surgical procedure may occlude several segmental arteries and promote collateral vessels enlargement, significantly altering the normal patterns of blood supply to the spinal cord [5].

Our meta-analysis was limited by the high amount of heterogeneity between the studies. However, the number of included studies and their large sample sizes mitigate this limitation. As cadaveric dissection is the gold standard for anatomical considerations, more cadaveric studies should assess prevalence of AKA, especially performed on subjects poorly represented in our meta-analysis, such as Africa, South America, and Oceania.

Because of the lower prevalence of AKA in radiological studies, surgeons should keep in mind that these results might be false negative. In this case, the risk of iatrogenic injury to the AKA during thoracolumbar surgical procedures is increased. More accurate imaging methods should be developed to assess the true prevalence of AKA.

To ensure spinal cord safety, preoperative AKA identification and its subsequent reconstruction or preservation are effective adjuncts for more secure protection of the spinal cord, along with other adequate management strategies.

## Conclusions

Our main findings revealed that the AKA was found to be present in the vast majority of the general population (84.6%), most often as a single vessel (87.4%) originating between T8 and L1 (89%) on the left side (76.6%). Based on our anatomical findings, we recommend that efforts should be made to identify and subsequently reconstruct or preserve the AKA to prevent postoperative neurological deficit due to spinal cord ischemia in vascular and endovascular surgical procedures in the thoracolumbar spinal cord.

## Electronic supplementary material

ESM 1 (DOC 64 kb)
